# Extensively Drug-Resistant Tuberculosis With Treatment-Limiting Mood Disorder: A Case of Complex Regimen Selection and Mental Health Challenges

**DOI:** 10.7759/cureus.93167

**Published:** 2025-09-25

**Authors:** Sahak Mkrtchyan

**Affiliations:** 1 Department of Tuberculosis, Yerevan State Medical University after Mkhitar Heratsi, Yerevan, ARM

**Keywords:** adverse effects, cycloserine, drug-induced hypersensitivity, extensive drug resistance, infectious disease control, multidisciplinary approach, psychiatric comorbidity, treatment of tb, tuberculosis (tb), xdr-tb

## Abstract

Extensively drug-resistant tuberculosis (XDR-TB) presents a significant therapeutic challenge due to resistance to both first-line and several second-line anti-tuberculosis drugs. Treatment planning becomes even more complex when psychiatric comorbidities and drug hypersensitivities further limit the therapeutic arsenal.

This case describes a 41-year-old man with a history of prior incarceration and incompletely treated multidrug-resistant tuberculosis, who presented with pulmonary symptoms and was subsequently diagnosed with XDR-TB. Drug susceptibility testing revealed extensive resistance, including to bedaquiline, clofazimine, and moxifloxacin. A pre-existing mood disorder necessitated the avoidance of neurotoxic agents such as cycloserine, and allergic reactions to several antibiotics led to further regimen modifications.

This case underscores the clinical difficulty of managing XDR-TB in the setting of psychiatric illness and drug intolerance. A multidisciplinary, individualized approach was crucial in designing a regimen that strikes a balance between antimicrobial effectiveness and mental health considerations. The patient demonstrated early clinical improvement and was discharged in stable condition with continued outpatient follow-up.

## Introduction

In 2023, tuberculosis (TB) re-emerged as the leading infectious cause of death globally, with approximately 8.2 million new cases and 1.25 million fatalities, the highest incidence recorded since 1995 [1]. TB presents a critical public health threat, particularly in resource-limited settings and among high-risk populations, including incarcerated individuals [2]. These populations often face systemic barriers to early diagnosis and continuity of care, thereby complicating TB control efforts. Additionally, clinical success for TB is significantly worse in individuals with mental health disorders compared to the general population [3].

Multidrug-resistant TB, defined as resistance to isoniazid and rifampin, represents a major challenge to global TB control. Extensively drug-resistant tuberculosis (XDR-TB) is an even more severe form of TB, characterized by resistance to at least isoniazid, rifampin, any fluoroquinolone, and at least one of the group A second-line drugs (the most effective second-line agents, such as bedaquiline or linezolid), as defined by the World Health Organization [4].

Globally, treatment success rates for XDR-TB remain low. For example, a recent systematic review reported a pooled treatment success rate of 44.2% among patients with XDR-TB [5]. Poor treatment outcomes contribute to the ongoing transmission of resistant strains, posing significant challenges to TB control programs worldwide.

Notably, several anti-TB drugs, particularly cycloserine and fluoroquinolones, are associated with psychiatric side effects such as depression, anxiety, and psychosis [6,7]. These adverse effects may worsen pre-existing mental health conditions and further complicate adherence and treatment outcomes. 

A case of XDR-TB is presented in a patient with multiple comorbidities, including mental health disorders and diabetes mellitus (DM). The management was complicated by drug resistance and treatment intolerance. This case highlights the real-world challenges in treating XDR-TB and underscores the importance of multidisciplinary care in vulnerable populations.

## Case presentation

A 41-year-old unemployed man with a history of insulin-dependent type 2 DM and previously treated multidrug-resistant pulmonary TB presented to a national TB center with a productive cough, chest pain, and generalized fatigue. He had been incarcerated abroad seven years prior, during which he was diagnosed with pulmonary TB approximately three years before presentation. His anti-TB treatment at that time was reportedly incomplete, with limited documentation available. Following release and return to his home country, he experienced progressive respiratory symptoms and was referred for specialized evaluation and care.

On admission, a comprehensive clinical examination was performed. The patient appeared in a moderately severe general condition. Vital signs were within normal limits, with normal oxygen saturation. Chest auscultation revealed decreased breath sounds bilaterally. There was no evidence of peripheral edema. The skin was clear and normally pigmented. Abdominal examination was unremarkable, with normal bowel and bladder habits reported.

Initial laboratory investigations demonstrated a hemoglobin level above the upper reference range, with red and white blood cell counts and platelet levels within normal limits. Inflammatory markers, including erythrocyte sedimentation rate, were not significantly elevated. Renal and hepatic function tests showed values within physiological limits. The coagulation profile was also unremarkable. Urinalysis revealed mild turbidity with normal specific gravity, absence of proteinuria and glycosuria, and minimal epithelial cell presence. All initial laboratory parameters are summarized in Table 1.

**Table 1:** Initial laboratory findings on admission

Parameter	Result	Reference range	Interpretation
Complete blood count			
Hemoglobin	184 g/L	130-170 g/L	Elevated
Red blood cells	6.14×10¹²/L	4.5-6.0×10¹²/L	Slightly elevated
White blood cells	9.75×10⁹/L	4.0-10.0×10⁹/L	Normal
Platelets	241×10⁹/L	150-400×10⁹/L	Normal
Erythrocyte sedimentation rate	3 mm/h	<20 mm/h	Low-normal
Blood biochemistry			
Creatinine	70 µmol/L	60-110 µmol/L	Normal
Urea	4.9 mmol/L	2.5-7.8 mmol/L	Normal
Alanine aminotransferase	15.9 U/L	<41 U/L	Normal
Aspartate aminotransferase	15.9 U/L	<40 U/L	Normal
Urinalysis			
Urine color	Yellow, turbid	-	Mildly abnormal appearance
Specific gravity	1.027	1.005-1.030	Normal
Glucose	None	Negative	Normal
Protein	None	Negative	Normal
Squamous epithelial cells	Few	0-5/hpf	Normal
Leukocytes	1-3/hpf	0-5/hpf	Normal

Initial microbiological examination of sputum samples demonstrated positive acid-fast bacilli on microscopy, and GeneXpert MTB/RIF confirmed rifampin resistance, indicating infection with *Mycobacterium tuberculosis*. Subsequent Xpert XDR testing identified resistance to isoniazid, fluoroquinolones, and kanamycin while confirming susceptibility to amikacin, capreomycin, and pretomanid.

Further profiling with culture-based drug susceptibility testing (DST) revealed additional resistance to bedaquiline, clofazimine, and moxifloxacin, establishing the diagnosis of XDR-TB. Despite this broad resistance profile, the isolate remained susceptible to ethambutol, pyrazinamide, amikacin, pretomanid, delamanid, and para-aminosalicylic acid.

**Figure 1:**
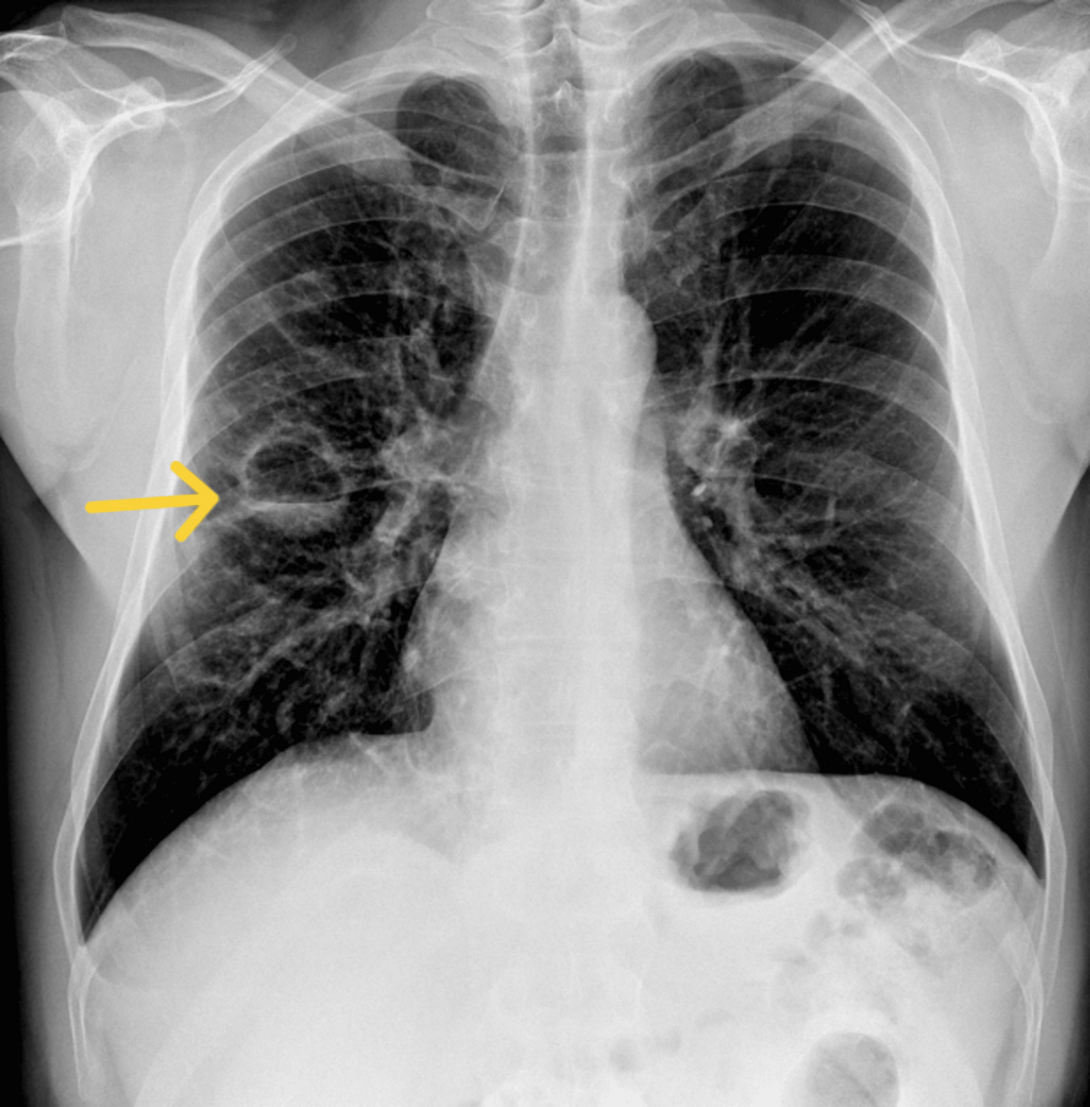
Chest X-ray (posteroanterior view) showing a 4 cm cavitary lesion with fibrous walls and a fluid level in the right upper lobe, indicated by the yellow arrow. Bilateral disseminated pulmonary foci are visible, more prominent on the right

Chest radiography revealed a 4 cm cavitary lesion with fibrous walls and a fluid level in the right upper lobe, accompanied by bilateral disseminated foci, more prominent in the right lung. The costophrenic angles were clear, and the pulmonary hila maintained a well-preserved structure. The corresponding chest X-ray is shown in Figure 1.

Abdominal ultrasonography identified diffuse hepatic lipodystrophy, a small gallbladder polyp, and mild urinary tract changes, including residual urine and bladder wall thickening. Cardiac evaluation revealed a normal sinus rhythm.

A multidisciplinary team, comprising specialists in pulmonology, endocrinology, ophthalmology, otolaryngology, and urology, collaboratively established the clinical diagnosis of XDR-TB with comorbid conditions, including insulin-dependent type 2 DM, non-proliferative diabetic retinopathy, diabetic distal sensorimotor polyneuropathy, right-sided mixed-type severe hearing loss, right-sided Eustachian tube dysfunction with nasal septum deviation, as well as chronic prostatitis and cystitis.

The initial treatment plan included bedaquiline, clofazimine, delamanid, imipenem, amoxicillin/clavulanate, and cycloserine. Cycloserine was temporarily discontinued following the Drug Committee's recommendation due to concerns about the patient's mental health. A psychiatric consultation, prompted by the patient's behavioral presentation, suggested the presence of a mood disorder. It was recommended to revise the treatment regimen to avoid exacerbating the psychiatric condition, leading to the discontinuation of cycloserine. Ethambutol was subsequently added to the treatment regimen.

After receiving the DST results, bedaquiline and clofazimine were discontinued due to resistance. Amikacin, linezolid, and prothionamide were then added to the treatment regimen. Imipenem and amoxicillin/clavulanate were later discontinued due to allergic reactions. Pyrazinamide was subsequently introduced.

In addition to the antimicrobial regimen, the patient received supportive therapies including betahistine, vinpocetine, diazepam, insulin therapy, detoxification therapy, and vitamin supplementation.

Two months after the initiation of therapy, the patient demonstrated clinical improvement. Sputum smears were negative for acid-fast bacilli on consecutive follow-up tests, and chest radiography revealed partial resolution of bilateral infiltrates with a significant reduction of the cavitary lesion. At discharge, vital signs were stable, although the patient reported dysuria and intermittent abdominal pain. The physical examination was grossly unremarkable except for diminished breath sounds. The patient was discharged with recommendations for outpatient follow-up, continuation of the modified anti-TB regimen, and management of DM and urological symptoms.

## Discussion

This case illustrates the clinical reality that managing XDR-TB extends far beyond simply following guideline-based regimens. The drug resistance pattern, which included resistance to bedaquiline, clofazimine, and fluoroquinolones, eliminated several of the most potent modern agents, forcing the use of less conventional combinations. Such patterns are relatively uncommon but represent a critical challenge, as they leave little margin for adverse reactions or drug intolerance.

In our case, the patient was initially diagnosed with multidrug-resistant TB during incarceration. Suboptimal adherence and incomplete treatment during this period likely facilitated the acquisition of further drug resistance, culminating in the development of XDR-TB [8].

Psychiatric comorbidity added a layer of complexity to the management of this patient. Cycloserine, often a key component of drug-resistant TB regimens, is associated with well-documented risks of mental health disorders [9]. Following consultation with a psychiatrist, the decision was made to discontinue cycloserine and replace it with ethambutol, which is generally considered to have a more favorable neuropsychiatric safety profile.

The patient's insulin-dependent DM represented yet another risk factor for poor TB outcomes [10]. DM impairs immune function, alters drug pharmacokinetics, and is associated with higher relapse rates. Coordinated involvement of endocrinology ensured optimized glycemic control, which is essential for maximizing treatment response.

Although carbapenems like imipenem are generally associated with a low risk of hypersensitivity reactions [11], allergic responses to beta-lactam antibiotics remain a significant clinical concern, with penicillins, such as amoxicillin/clavulanate, being among the most common causes of drug allergies [12]. In this case, allergic reactions to both imipenem and amoxicillin/clavulanate further limited the treatment options, leading to the decision to replace these drugs with pyrazinamide as one of the remaining effective agents. This highlights the importance of prompt recognition and substitution to maintain regimen efficacy in the management of XDR-TB.

Despite these challenges, early sputum conversion and radiographic improvement were achieved, reflecting the benefits of individualized therapy supported by multidisciplinary input from pulmonology, psychiatry, endocrinology, and infectious disease specialists. This outcome reinforces that even in highly constrained situations, dynamic regimen adjustments, close clinical monitoring, and proactive management of comorbidities can yield favorable short-term results. Long-term follow-up will be essential to assess sustained cure and prevent relapse.

## Conclusions

Managing XDR-TB in patients with psychiatric comorbidities presents significant clinical challenges. This case highlights the importance of an individualized, multidisciplinary approach to balance effective antimicrobial therapy with mental health considerations. Careful regimen selection, informed by DST and psychiatric evaluation, alongside close clinical monitoring, is essential to optimize treatment outcomes. Ongoing follow-up remains crucial to ensure sustained cure and prevent relapse in this complex patient population.
